# Structure-based discovery and experimental validation of HIT101481851 as a potential PKMYT1 inhibitor for pancreatic cancer

**DOI:** 10.3389/fphar.2025.1605741

**Published:** 2025-06-18

**Authors:** Ting Wang, Jingyu Wang, Gongxiong Yao, Hongchao Zhang, Chenghui Song, Xueren Ao

**Affiliations:** ^1^The Third Clinical Medical College of Guangzhou University of Chinese Medicine, Guangzhou, China; ^2^ The Third Affiliated Hospital, Guangzhou University of Chinese Medicine, Guangzhou, China

**Keywords:** PKMYT1, pancreatic cancer, virtual screening, molecular dynamics, experimental validation

## Abstract

PKMYT1 is a validated therapeutic target in pancreatic cancer due to its critical role in controlling the G2/M transition of the cell cycle. In this study, a structure-based drug discovery pipeline was implemented to identify novel PKMYT1 inhibitors with high binding stability and anticancer potential. Pharmacophore models were constructed from four PKMYT1 co-crystal structures, and virtual screening was performed against a large compound library. Through molecular docking and intersection analysis, five consensus high-affinity compounds were identified, among which HIT101481851 demonstrated the most favorable binding characteristics. Molecular dynamics simulations confirmed its stable interactions with key residues such as CYS-190 and PHE-240 across multiple PKMYT1 conformations. ADMET predictions indicated good gastrointestinal absorption, acceptable drug-likeness, and low risk of off-target reactivity. Furthermore, *in vivo* experiments showed that HIT101481851 inhibited the viability of pancreatic cancer cell lines in a dose-dependent manner while exhibiting lower toxicity toward normal pancreatic epithelial cells. These results suggest that HIT101481851 is a promising lead compound for the development of PKMYT1-targeted therapeutics in pancreatic cancer.

## 1 Introduction

Pancreatic ductal adenocarcinoma (PDAC) remains one of the most aggressive and lethal malignancies worldwide, with a dismal prognosis and limited therapeutic options. Recent epidemiological data indicate that PDAC is responsible for over 496,000 new cases and 466,000 deaths annually on a global scale, placing it among the top causes of cancer-related mortality (Siegel et al., 2024). The persistent rise in incidence and mortality is attributed to multiple risk factors including population aging, smoking, obesity, and hereditary susceptibility (Cai et al., 2021). Despite ongoing advances in diagnostics and treatment, the 5-year survival rate remains below 12%, primarily due to late-stage diagnosis, pronounced metastatic potential, and resistance to conventional chemotherapy (Zeng et al., 2024). Moreover, PDAC is characterized by a highly complex and immunosuppressive tumor microenvironment (TME), which features dense stroma, tumor-associated macrophages (Uttamsingh et al., 2015), and myeloid-derived suppressor cells (MDSCs), further hindering effective drug delivery and immunotherapeutic success (Herting et al., 2021). These challenges emphasize the urgent need to explore novel therapeutic targets and design more effective antitumor strategies.

Protein kinase membrane-associated tyrosine/threonine 1 (PKMYT1), a member of the WEE family of kinases, has recently emerged as a potential druggable target in multiple cancers including PDAC (Yang et al., 2024). PKMYT1 is distinguished from its nuclear counterpart WEE1 by its cytoplasmic localization (Tomović Pavlović et al., 2024) and its ability to phosphorylate CDK1 at both THR-14 and TYR-15, thereby halting mitotic entry in response to replication stress (Asquith et al., 2020). Overexpression of PKMYT1 in PDAC correlates strongly with poor prognosis, and its inhibition has been shown to induce mitotic catastrophe and apoptosis in cancer cells dependent on the G2/M checkpoint, while sparing normal cells. These properties render PKMYT1 an attractive and selective therapeutic target (Wang S. et al., 2024).

The past few years have witnessed the discovery of several potent PKMYT1 inhibitors. Among them, RP-6306 developed by Repare Therapeutics is the first orally bioavailable and selective PKMYT1 inhibitor that has shown robust antitumor activity in preclinical models and is currently under evaluation in phase II clinical trials for patients with advanced cancer (Szychowski et al., 2022). Building on the structural insights of RP-6306, researchers have pursued structure-based design approaches to expand the chemical space of PKMYT1 inhibitors. A recent study reported the development of a new class of 2-amino-[1,1′-biphenyl]-3-carboxamide derivatives that selectively inhibit PKMYT1 with nanomolar potency (Wang C. et al., 2024). For instance, compound 8ma demonstrated an IC_50_ value of 16.5 nM and exhibited excellent selectivity over WEE1. Crystallographic analysis confirmed that its enhanced potency was due to specific hydrogen bonding interactions with key residues Asp251 and Tyr121 of the ATP-binding domain of PKMYT1. These efforts not only validate PKMYT1 as a clinically relevant target but also provide promising chemical scaffolds for further optimization.

In this study, we employed a combination of computer-aided drug design (CADD) and experimental validation to screen and identify novel PKMYT1 inhibitors with potential application in pancreatic cancer therapy. In the initial virtual screening phase, we utilized the new class of 2-amino-[1,1′-biphenyl]-3-carboxamide derivatives as a reference and explored multiple PKMYT1 crystal structures. Based on pharmacophore modeling and molecular docking, we systematically screened chemical libraries to identify compounds with favorable binding profiles and selectivity potential. This integrative approach aimed to provide new insights into PKMYT1-targeted drug discovery and offer promising leads for future translational research in PDAC treatment.

## 2 Materials and methods

### 2.1 Protein and ligand preparation

Obtained from the Protein Data Bank, the crystal structure of PKMYT1 with the reference inhibitors (PDB ID 8ZTX, 8ZU2, 8ZUD, and 8ZUL) (Wang C. et al., 2024) displays a resolution of 1.88 Å. Through the Schrodinger 2024-1 suite’s protein preparation wizard (Schrödinger Release 2024-1: Protein Preparation, Schrödinger, LLC, New York, NY, 2024.) (Sastry et al., 2013) and prime module, hydrogen atoms are added, missing loops filled, termini capped, charge states adjusted, and inappropriate H-bond orders fixed. Various steric strains and heavy atoms up to 0.3 Å were eliminated using the restrained energy minimization OPLS 2005 force field (Jorgensen et al., 1996). All the compound structures (1.64 M) sourced from the TargetMol natural compound library were prepared by the LigPrep module (Greenwood et al., 2010). The pH range for this module was set as 7.0 ± 2.0. The OPLS4 force field was used for structural validation and energy minimization (Lu et al., 2021). The binding region of the pyrimidine derivative was identified as the target site, and a corresponding grid was created.

### 2.2 Pharmacophore-based screening

Pharmacophore modeling was conducted using the Phase module (Phase, Schrödinger, LLC, New York, NY, 2024) in Schrödinger’s Maestro suite (Dixon et al., 2006b; Dixon et al., 2006a), based on ligand conformations extracted directly from co-crystal structures of PKMYT1. Specifically, four high-resolution PKMYT1-ligand complex structures (PDB IDs: 8ZTX, 8ZU2, 8ZUD, and 8ZUL) were selected to represent diverse binding conformations and key pharmacophoric features within the ATP-binding pocket. The bound ligands were individually extracted and aligned to identify shared interaction patterns. From this analysis, representative pharmacophore models were generated, incorporating critical features such as hydrogen bond acceptors/donors, aromatic rings, and hydrophobic centers as observed in the active binding site. The resulting pharmacophore models were then used to screen compound libraries for potential hits exhibiting similar spatial and chemical characteristics. Top-scoring compounds from the pharmacophore-based screening were selected for subsequent structure-based molecular docking.

### 2.3 Structure-based molecular docking

The compounds retrieved from pharmacophore-based screening were subjected to structure-based molecular docking using the Glide module in Schrödinger (Friesner et al., 2004; Repasky et al., 2012; Cross et al., 2009). Protein-ligand complexes of PKMYT1 (PDB IDs: 8ZTX, 8ZU2, 8ZUD, and 8ZUL) were used as docking templates. For each structure, the co-crystallized ligand was retained to define the center of the docking grid, ensuring that the docking box encompassed the biologically relevant binding site. Protein structures were preprocessed using the Protein Preparation Wizard, including protonation state adjustment, optimization of hydrogen bonding networks, and energy minimization.

Docking was performed in a hierarchical manner using three sequential modes: high-throughput virtual screening (HTVS), standard precision (SP), and extra precision (XP) (Friesner et al., 2006). HTVS was applied to rapidly filter large libraries, SP was used for intermediate scoring and pose refinement, and XP was employed for accurate docking and scoring of top-ranked compounds. Redocking of the native ligand was conducted to validate the docking protocol (Agu et al., 2023). Selected compounds with favorable Glide scores and binding poses were further evaluated in subsequent molecular dynamics simulations and MM-GBSA binding free energy calculations.

### 2.4 Molecular dynamics simulation analysis

To investigate the dynamic behavior of the protein–ligand complexes and the stability of their interactions over time, molecular dynamics (MD) simulations were performed on 20 selected PKMYT1–ligand complexes along with four reference complexes. All simulations were conducted using Desmond (Desmond, Schrödinger, LLC, New York, NY, 2024). Each system underwent a 1-microsecond (1 μs) simulation to comprehensively capture conformational fluctuations and interaction profiles throughout the simulation period.

Prior to simulation, the protein structures were prepared using Desmond’s Protein Preparation Wizard. This included assigning proper bond orders, adding hydrogen atoms, modeling any missing loops or side chains, adjusting protonation states at physiological pH, and performing restrained energy minimization. Water molecules within the binding pocket were evaluated and retained only if deemed functionally relevant. The systems were solvated with explicit water using the TIP3P model, and counterions were introduced to neutralize the total charge of each complex (Mark and Nilsson, 2001).

All atoms in the systems were parameterized using the OPLS4 force field to ensure reliable representation of physical interactions. Initial energy minimization was performed to remove steric clashes, followed by a two-stage equilibration protocol: 100 ps under NVT ensemble (constant volume and temperature), and 10 ns under NPT ensemble (constant pressure and temperature), maintaining 300 K and 1 atm. The Nose–Hoover chain thermostat (Barrett and Minus, 2025) and Martyna–Tobias–Klein barostat (Rogge et al., 2015) were employed for temperature and pressure control, respectively (Tripathi et al., 2025).

Following equilibration, each system was subjected to a production MD simulation for 1 μs. Periodic boundary conditions were applied, and integration steps were performed at 2 fs intervals. Long-range electrostatics were calculated using the particle mesh Ewald (PME) method, with a short-range cutoff distance of 9.0 Å (Petersen, 1995). Trajectory analysis was carried out using Desmond’s built-in tools, focusing primarily on root mean square deviation (RMSD) and protein–ligand interaction mapping to evaluate complex stability and binding behavior over time.

### 2.5 Cell culture

Three human pancreatic cancer cell lines—MiaPaCa-2, BxPC-3, and PANC-1—and a non-tumorigenic human pancreatic ductal epithelial cell line hTERT-HPNE were used in this study. MiaPaCa-2 and PANC-1 cells were cultured in Dulbecco’s Modified Eagle Medium (DMEM; Gibco, United States) supplemented with 10% fetal bovine serum (FBS; Gibco), 1% penicillin-streptomycin (100 U/mL penicillin and 100 μg/mL streptomycin), and maintained at 37°C in a humidified atmosphere containing 5% CO_2_. BxPC-3 cells were maintained in RPMI-1640 medium (Gibco) supplemented with 10% FBS and 1% penicillin-streptomycin under the same incubation conditions (Zhao et al., 2024; Chiu et al., 2021).

The non-tumorigenic hTERT-HPNE cell line was cultured in a 1:1 mixture of DMEM and Medium M3 Base (InCell, United States) supplemented with 10% FBS, 5 ng/mL epidermal growth factor (EGF; PeproTech), 10 mM HEPES, 2 mM L-glutamine, and 1% penicillin-streptomycin. All cell lines were routinely tested and confirmed to be free of *mycoplasma* contamination (Zhao et al., 2024).

### 2.6 Cell viability assay (CCK-8)

The antiproliferative effects of HIT101481851 on human pancreatic cancer cell lines (MiaPaCa-2, BxPC-3, and PANC-1) and the non-tumorigenic pancreatic ductal epithelial cell line (hTERT-HPNE) were evaluated using the Cell Counting Kit-8 (CCK-8; Dojindo, Japan). HIT101481851 was purchased from TargetMol (Boston, MA, United States) and dissolved in DMSO to prepare a stock solution.

Cells were seeded in 96-well plates at a density of 4 × 10^3^ cells per well in 100 μL of complete growth medium and allowed to adhere overnight. The next day, cells were treated with HIT101481851 at final concentrations of 0, 1, 5, 10, 20, 40, 80, and 100 μM for 24 h. After treatment, 10 μL of CCK-8 solution was added to each well, and the plates were incubated for 2 h at 37°C.

The absorbance at 450 nm was measured using a microplate reader (BioTek, United States). Cell viability was calculated as a percentage of the control (0 μM group). Each experiment was performed in triplicate, and results were presented as mean ± standard deviation (Novikova et al., 2018).

## 3 Results

### 3.1 Pharmacophore modeling and virtual screening based on PKMYT1 co-crystal structures

**FIGURE 1:**
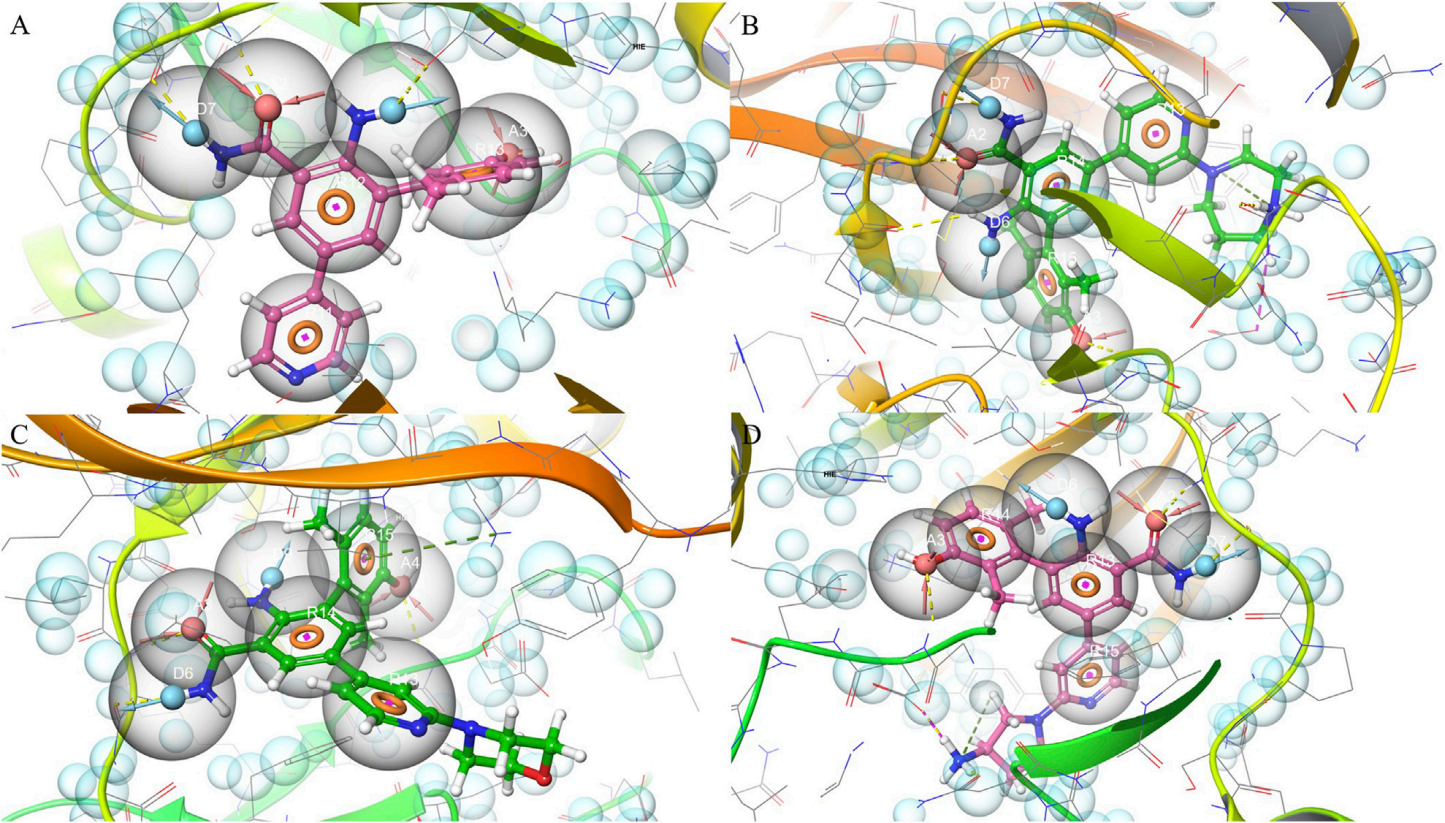
Pharmacophore modeling based on four PKMYT1 crystal structures. **(A)** 8ZTX; **(B)** 8ZU2; **(C)** 8ZUD; **(D)** 8ZUL.

Pharmacophore features were extracted from four ligand-bound crystal structures of PKMYT1 (PDB IDs: 8ZTX, 8ZU2, 8ZUD, and 8ZUL), capturing conserved interaction patterns within the binding pocket. In 8ZTX, as shown in Figure 1A, key features included CYS-190 (A2), THR-187 (D6), and ASP-251 (A3). Notably, ASP-251 is a known Mg^2+^-binding residue, indicating that the compound may mimic coordination at the metal-binding site. The 8ZU2 structure revealed a similar interaction pattern, as shown in Figure 1B, comprising CYS-190 (A2), TYR-136 (D7), and ASP-251 (A3), with additional contacts at GLU-188, TYR-121, and ALA-237. Among these, TYR-136 lies within the ATP-binding region (residues 116–124), suggesting potential competitive interactions. In 8ZUD, as shown in Figure 1C, the model incorporated LEU-189 (A3), CYS-190 (D6), and a ring interaction involving LYS-139 (R15); the latter corresponds to a critical ATP-binding residue, further supporting ligand engagement in the nucleotide-binding site. The pharmacophore also included an undefined acceptor (A4). In 8ZUL, as shown in Figure 1D, ASP-251 (A3) and CYS-190 (A2, D7) were consistently retained, while additional interactions with TYR-121 and ASN-238 were observed. ASN-238 is proximal to the Mg^2+^-binding region, reinforcing the potential for metal-chelating interactions.

Across all complexes, CYS-190 and ASP-251 emerged as conserved pharmacophoric anchors, reflecting their roles in ligand stabilization and potential interference with the catalytic or cofactor-binding machinery. The spatial convergence of donor, acceptor, and ring features across distinct scaffolds provided a structurally coherent model applicable to structure-based drug discovery.

These pharmacophore models were subsequently employed in a structure-based virtual screening of a chemical library containing approximately 1.64 million compounds. Each model was independently applied to identify ligands exhibiting spatial and chemical complementarity to the defined interaction features. Using the 8ZTX-based model, 54,427 compounds corresponding to 107,236 conformers were retrieved. The 8ZU2 model yielded 53,964 compounds and 97,385 conformers. For the 8ZUD model, 54,476 compounds and 79,610 conformers were matched, while the 8ZUL model identified 54,317 compounds and 82,535 conformers. The screening results highlight the structural specificity and mutual complementarity of the pharmacophore models, forming a robust foundation for downstream hit prioritization and molecular docking studies.

### 3.2 Refinement of pharmacophore-based hits and identification of consensus high-affinity compounds

To refine the pharmacophore-derived candidates, all conformers obtained from the virtual screening step were subjected to molecular docking against their corresponding PKMYT1 crystal structures. To eliminate redundancy, only the highest-scoring conformer was retained for each unique compound.

**FIGURE 2:**
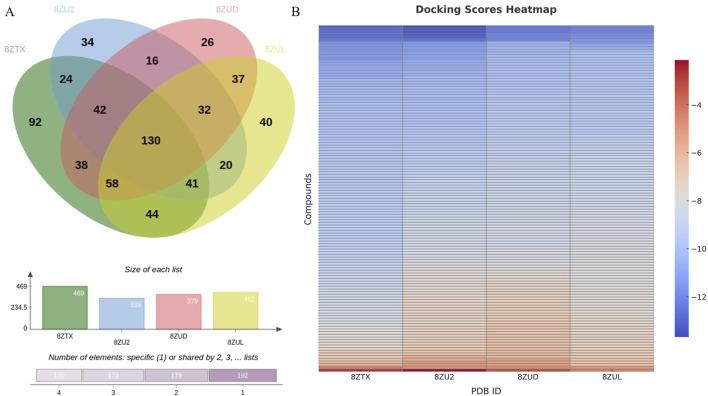
Intersection analysis of hit compounds and binding evaluation of the 130 consensus compounds. **(A)** Venn diagram illustrating the overlap of unique compounds identified through docking of conformers generated from four pharmacophore models (8ZTX, 8ZU2, 8ZUD, 8ZUL), resulting in 130 consensus compounds. **(B)** Heatmap visualization of docking scores for these 130 compounds docked into all four crystal structures. Compounds exhibiting docking scores better than the redocking thresholds of the native ligands [−12.0702 (8ZTX), −13.0052 (8ZU2), −12.2000 (8ZUD), −11.5608 (8ZUL)] were retained for further investigation.

Docking of 107,236 conformers generated by the 8ZTX-based pharmacophore yielded 469 unique compounds. Similarly, docking of 97,385, 79,610, and 82,535 conformers from the 8ZU2, 8ZUD, and 8ZUL models resulted in 339, 379, and 402 unique compounds, respectively. The intersection of these four compound sets was analyzed using a Venn diagram (Figure 2A), identifying 130 consensus compounds that were commonly retained across all four pharmacophore models.

These 130 overlapping hits were subsequently docked into all four crystal structures to evaluate their binding profiles. Docking scores were visualized in a heatmap (Figure 2B), and native ligand redocking scores were used as selection thresholds: −12.0702 (8ZTX), −13.0052 (8ZU2), −12.2000 (8ZUD), and −11.5608 (8ZUL). Compounds with docking scores surpassing all four thresholds were retained for further consideration.

This rigorous selection process identified five high-confidence candidate compounds: HIT105013765, HIT104637306, HIT103795627, HIT105869298, and HIT101481851. Their docking scores across the four crystal structures are summarized in Table 1. All five compounds consistently exhibited docking affinities superior to those of the respective co-crystallized ligands, indicating favorable binding across multiple PKMYT1 conformations.

**TABLE 1:** Docking scores (kcal/mol) of the top five consensus compounds against four PKMYT1 crystal structures.

Compound ID	8ZTX	8ZU2	8ZUD	8ZUL
HIT105013765	−13.181	−13.675	−12.546	−12.225
HIT104637306	−13.006	−13.509	−12.529	−12.158
HIT103795627	−12.827	−13.432	−12.471	−12.136
HIT105869298	−12.782	−13.366	−12.203	−11.84
HIT101481851	−12.692	−13.104	−12.201	−11.812

**FIGURE 3:**
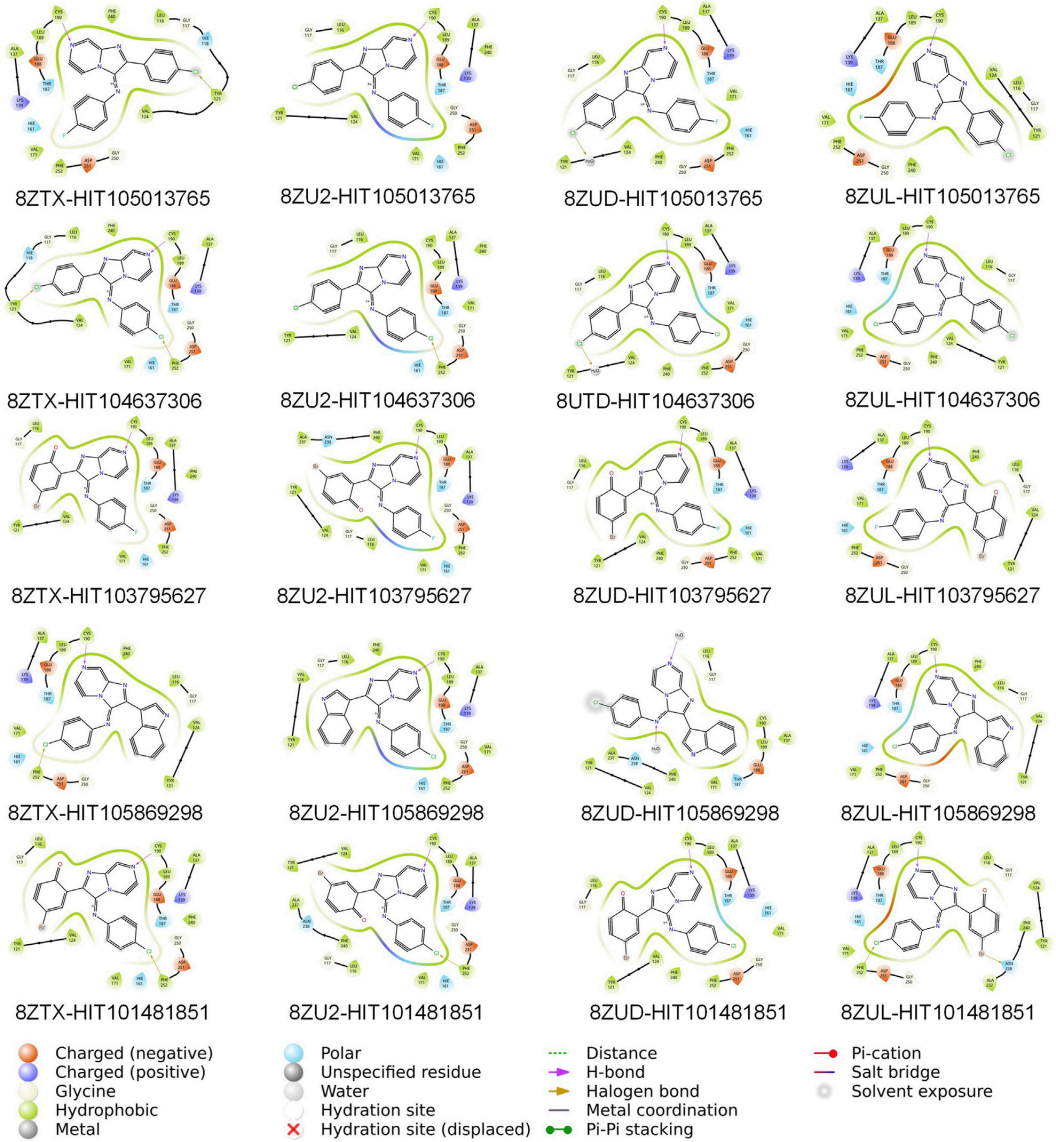
Two-dimensional interaction diagrams of twenty consensus compounds docked into four PKMYT1 crystal structures. From left to right, the compounds are arranged as: HIT105013765, HIT104637306, HIT103795627, HIT105869298, and HIT101481851. From top to bottom, the protein structures are ordered as: 8ZTX, 8ZU2, 8ZUD, and 8ZUL. Each panel illustrates key binding interactions between the ligand and the protein, including hydrogen bonds, hydrophobic contacts, and other relevant features.

Further inspection of protein–ligand interactions revealed that CYS-190, a residue recurrently identified in pharmacophore features, served as a dominant anchoring site across all hits, as shown in Figure 3. HIT105013765 interacted with CYS-190 in all four structures and formed an additional contact with TYR-121 in 8ZTX. HIT104637306 established interactions with CYS-190 in three structures and also contacted TYR-121 and PHE-252 in 8ZTX, and PHE-252 in 8ZU2. HIT103795627 consistently bound to CYS-190 in all conformations. HIT105869298 engaged CYS-190 in three structures and interacted with PHE-252 in 8ZTX. Notably, HIT101481851 displayed the most extensive interaction network, forming contacts with CYS-190 across all four structures, with additional interactions involving PHE-252 in three complexes and ASN-238 in 8ZUL. The latter residue lies proximal to the Mg^2+^-binding site, suggesting a potential for cofactor-interfering activity.

### 3.3 Molecular dynamics evaluation of ligand binding stability across PKMYT1 conformations

**FIGURE 4:**
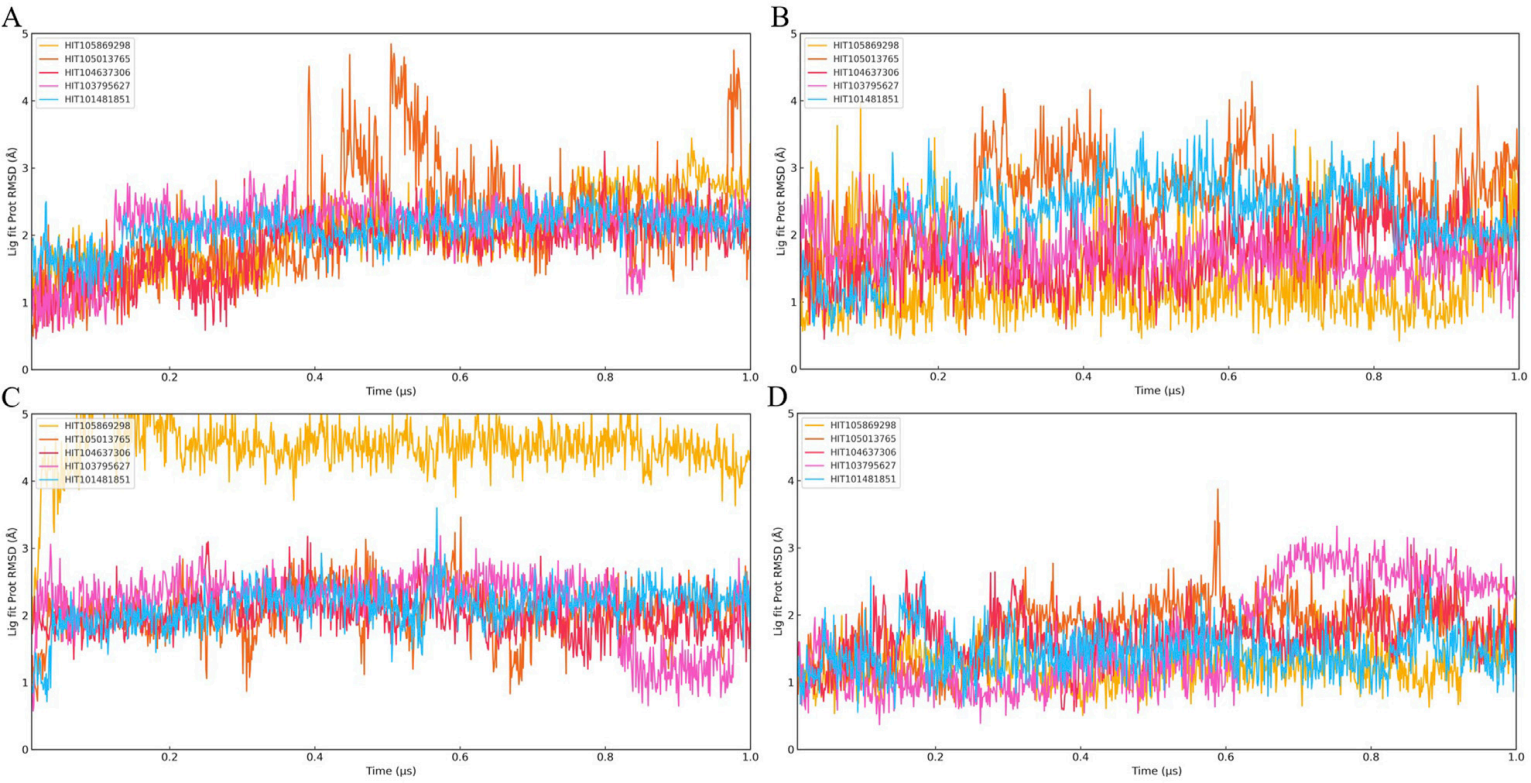
Time evolution of Ligand Fit on Protein RMSD over 1 μs molecular dynamics simulations for twenty PKMYT1-ligand complexes. **(A)** RMSD profiles of five compounds bound to 8ZU2; **(B)** five compounds bound to 8ZUD; **(C)** five compounds bound to 8ZTX; **(D)** five compounds bound to 8ZUL. The five compounds shown in each panel are ordered as follows: HIT105013765, HIT104637306, HIT103795627, HIT105869298, and HIT101481851. These results were used to evaluate the binding stability of ligands under different protein conformations.

To assess the conformational stability and binding persistence of the five candidate compounds, molecular dynamics (MD) simulations were conducted using four PKMYT1 crystal structures (8ZTX, 8ZU2, 8ZUD, and 8ZUL). Ligand RMSD trajectories were monitored throughout the simulations to evaluate structural retention and dynamic adaptability. The results are summarized in Figures 4A–D.

Among the five compounds, HIT105013765 consistently exhibited the greatest degree of conformational fluctuation. In multiple systems, including 8ZTX and 8ZUD, the RMSD values exceeded 4 Å and failed to stabilize over time, indicating an unstable binding mode with no clear convergence. Similar trends were observed in 8ZU2 and 8ZUL, where no distinct plateau was formed and significant structural deviations persisted throughout the simulation, suggesting high flexibility and poor conformational retention.

HIT104637306 demonstrated moderate stability, with performance varying across different protein conformations. In 8ZTX, its RMSD initially fluctuated but gradually stabilized, with only transient peaks exceeding 3 Å. The compound remained relatively stable in 8ZU2 and 8ZUD, where RMSD values were consistently low with minor perturbations. However, in the 8ZUL system, pronounced fluctuations and the absence of a defined plateau suggested reduced structural compatibility and binding stability in this conformation.

HIT103795627 maintained a well-defined and persistent binding mode in most systems. In 8ZTX and 8ZU2, RMSD values remained below 3 Å with minimal fluctuations, indicating excellent structural retention. In 8ZUD, the RMSD profile revealed two discrete low-energy states, suggesting a conformational shift between energetically favorable binding poses. A similar observation was made in 8ZUL, where the trajectory exhibited three distinguishable phases, reflecting conformational polymorphism while remaining within a constrained and stable range.

HIT105869298 showed variable behavior across different structures. In 8ZTX, it experienced significant RMSD fluctuations exceeding 3 Å and lacked convergence. The ligand displayed moderate instability in 8ZU2 and 8ZUD, with occasional spikes and an absence of consistent plateaus. In contrast, the simulation in 8ZUL revealed a low and stable RMSD trajectory, indicating a more favorable binding configuration under this conformation.

HIT101481851 consistently demonstrated the most favorable dynamic behavior among all candidates. In all four systems, the ligand maintained RMSD values below 3 Å with smooth, stable trajectories and only minor transient deviations. Notably, the simulation in 8ZUL displayed a particularly flat RMSD curve, while 8ZUD and 8ZTX exhibited gentle fluctuations without significant drift, suggesting strong conformational retention with controlled flexibility. These characteristics reflect a dynamically stable binding mode with high adaptability and low structural perturbation.

Collectively, these results indicate that HIT101481851 possesses the most robust conformational stability and binding persistence across diverse PKMYT1 conformations, followed by HIT103795627, which also exhibited consistent structural retention. In contrast, HIT105013765 and HIT104637306 displayed varying degrees of instability and are less suitable for further development. HIT105869298 showed structure-dependent behavior, with limited potential.

It is noteworthy that none of the five compounds exhibited clear RMSD convergence in the 8ZU2 system, with all trajectories lacking stable plateau phases. This suggests either an intrinsic flexibility or structural instability of the 8ZU2 conformation, or a general incompatibility of this protein state with the ligand chemotypes studied. Based on this observation, subsequent analyses were focused exclusively on the remaining three crystal structures (8ZTX, 8ZUD, and 8ZUL), and the 8ZU2 model was excluded from further evaluation of compound–protein interactions.

### 3.4 Conserved interaction profiles of HIT101481851 across PKMYT1 conformations

**FIGURE 5:**
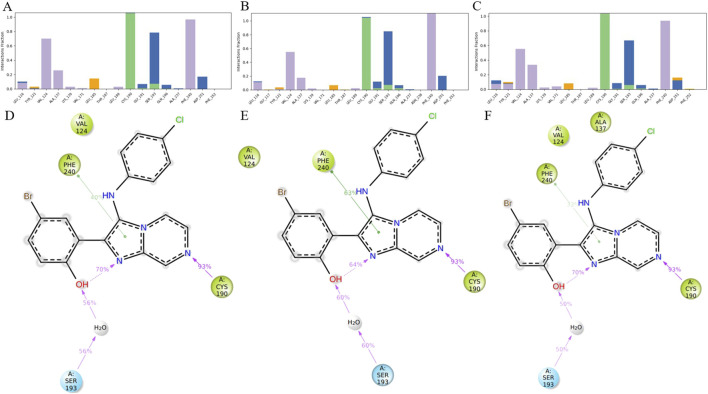
Analysis of ligand–residue interactions within PKMYT1 complexes. **(A–C)** Interaction frequencies between the ligand and residues in 8ZTX, 8ZUD, and 8ZUL crystal structures, respectively. **(D–F)** Atomic-level interaction breakdowns showing only contacts that occurred in more than 30% of the simulation time (0–1,000 ns). Due to the presence of multiple atoms capable of forming the same type of interaction (e.g., in ARG or SER), individual residues may exhibit cumulative interaction frequencies exceeding 100%.

To further elucidate the binding mechanism of HIT101481851, interaction fingerprint analyses were performed based on MD trajectories of the 8ZTX, 8ZUD, and 8ZUL crystal structures. The resulting interaction profiles are illustrated in Figures 5A–C and demonstrate a high degree of consistency across all three conformations.

In the 8ZTX structure, HIT101481851 primarily engages with four residues—VAL-124, PHE-240, CYS-190, and SER-193—all exhibiting interaction fractions greater than 0.3. VAL-124 and PHE-240 contribute through persistent hydrophobic contacts, characteristic of alkyl and aromatic side chains, with contact distances maintained below 4.5 Å. CYS-190 forms stable hydrogen bonds with the ligand, specifically involving side-chain donor interactions, as defined by a donor–acceptor distance ≤2.5 Å, a donor angle ≥120°, and an acceptor angle ≥90°. SER-193 mediates its interaction mainly via water-bridged hydrogen bonds, which satisfy geometric criteria of ≤2.8 Å distance, donor angle ≥110°, and acceptor angle ≥90°, and also contributes partially through direct hydrogen bonding.

A nearly identical interaction profile is observed in the 8ZUD structure, with the same four residues participating as primary interaction partners. This consistent pattern indicates a conserved binding mode that is resilient to moderate structural variations in PKMYT1. In the 8ZUL system, all four residues—VAL-124, PHE-240, CYS-190, and SER-193—remain active binding partners. Additionally, ALA-137 exhibits an interaction fraction slightly exceeding 0.3. As shown in Figures 5A–C, this contact is unique to 8ZUL. However, historical interaction data suggest that ALA-137 displayed near-threshold interaction frequencies (∼0.3) in the other two complexes as well, implying that its detection in 8ZUL may fall within statistical variation rather than indicating a functionally significant difference.

The interaction breakdown in Figures 5D–F provides a detailed schematic view of ligand–residue contacts at the atomic level. Only interactions occurring in more than 30% of the simulation time (0–1,000 ns) are shown. Notably, certain residues, particularly those with flexible or functionally rich side chains (e.g., ARG or SER), may contribute to multiple simultaneous interactions of the same type. Therefore, individual residues may show cumulative interaction fractions exceeding 100%, due to the presence of multiple atoms (e.g., four hydrogen bond donors in ARG) forming independent hydrogen bonds with the same ligand atom. This representation offers a quantitative view of interaction persistence and highlights the dominant chemical contributions sustaining ligand binding.

Across all three systems, the observed interactions span a range of biophysically meaningful categories, including hydrogen bonding, water bridges, and hydrophobic contacts. No significant halogen bonds or ionic interactions (defined as electrostatic contacts between oppositely charged atoms within 3.7 Å and not classified as hydrogen bonds) were detected in these simulations. The hydrogen bonding subtypes were further categorized into backbone or side-chain donors and acceptors, based on geometric analysis. No halogen atoms were present in HIT101481851, thus precluding halogen bonding in this case.

Taken together, HIT101481851 exhibits a remarkably conserved and robust binding pattern with PKMYT1, maintained across three distinct crystal structures. The consistency of high-frequency contacts—particularly with VAL-124, PHE-240, CYS-190, and SER-193—reinforces the ligand’s conformational adaptability and its low dependency on protein structural variation. This interaction stability further substantiates HIT101481851 as the most promising compound for continued structure-based optimization and functional validation.

### 3.5 ADMET profiling and drug-likeness evaluation of HIT101481851

To assess the pharmacokinetic suitability of HIT101481851, a comprehensive ADMET (Absorption, Distribution, Metabolism, Excretion, and Toxicity) analysis was conducted using the SWISS ADME online platform, detailed in Supplementary Table 1. The compound demonstrated favorable gastrointestinal absorption (GI absorption = High) and was predicted not to be a substrate of P-glycoprotein (P-gp), which may reduce the risk of efflux-mediated bioavailability loss and contribute to improve *in vivo* absorption.

Despite its poor aqueous solubility (Log S ranging from −6.11 to −7.60, categorized as “poorly soluble”), the compound exhibited moderate lipophilicity (Consensus Log Po/w = 3.99), which falls within the optimal range for drug-like molecules and supports the potential for efficient passive membrane permeability.

Regarding tissue distribution, the compound was predicted to be blood-brain barrier (BBB) permeant, suggesting possible application in central nervous system (CNS)–related indications. However, this property also raises concerns about potential off-target effects in the CNS, warranting further safety evaluation. The predicted skin permeability (Log Kp = −5.15 cm/s) was within a moderate-to-low range, indicative of limited transdermal absorption.

From a metabolic standpoint, HIT101481851 was predicted to inhibit multiple cytochrome P450 isoforms, including CYP1A2, CYP2C19, CYP2C9, CYP2D6, and CYP3A4. Such broad-spectrum CYP inhibition suggests a potential risk for drug–drug interactions, underscoring the need for experimental validation of its metabolic stability and enzyme specificity in subsequent studies.

In terms of drug-likeness, the compound satisfied major filtering rules, including those of Lipinski, Ghose, Veber, and Egan, with only a minor violation of the Muegge rule, attributed to an XLOGP3 value slightly exceeding five. The bioavailability score was calculated at 0.55, reflecting a moderate likelihood of oral bioavailability. Importantly, the compound triggered no alerts in either the PAINS or Brenk filters, indicating a low propensity for nonspecific reactivity or false-positive outcomes in high-throughput screening campaigns.

Although the compound did not fully meet the criteria for “lead-likeness” due to its molecular weight exceeding 350 Da and a relatively high XLOGP3 (>3.5), the synthetic accessibility score of 3.16 suggests moderate feasibility for chemical synthesis and optimization. These attributes collectively support its potential as a viable starting point for further medicinal chemistry refinement.

### 3.6 *In vitro* cytotoxicity evaluation of HIT101481851 in pancreatic cancer cells

**FIGURE 6:**
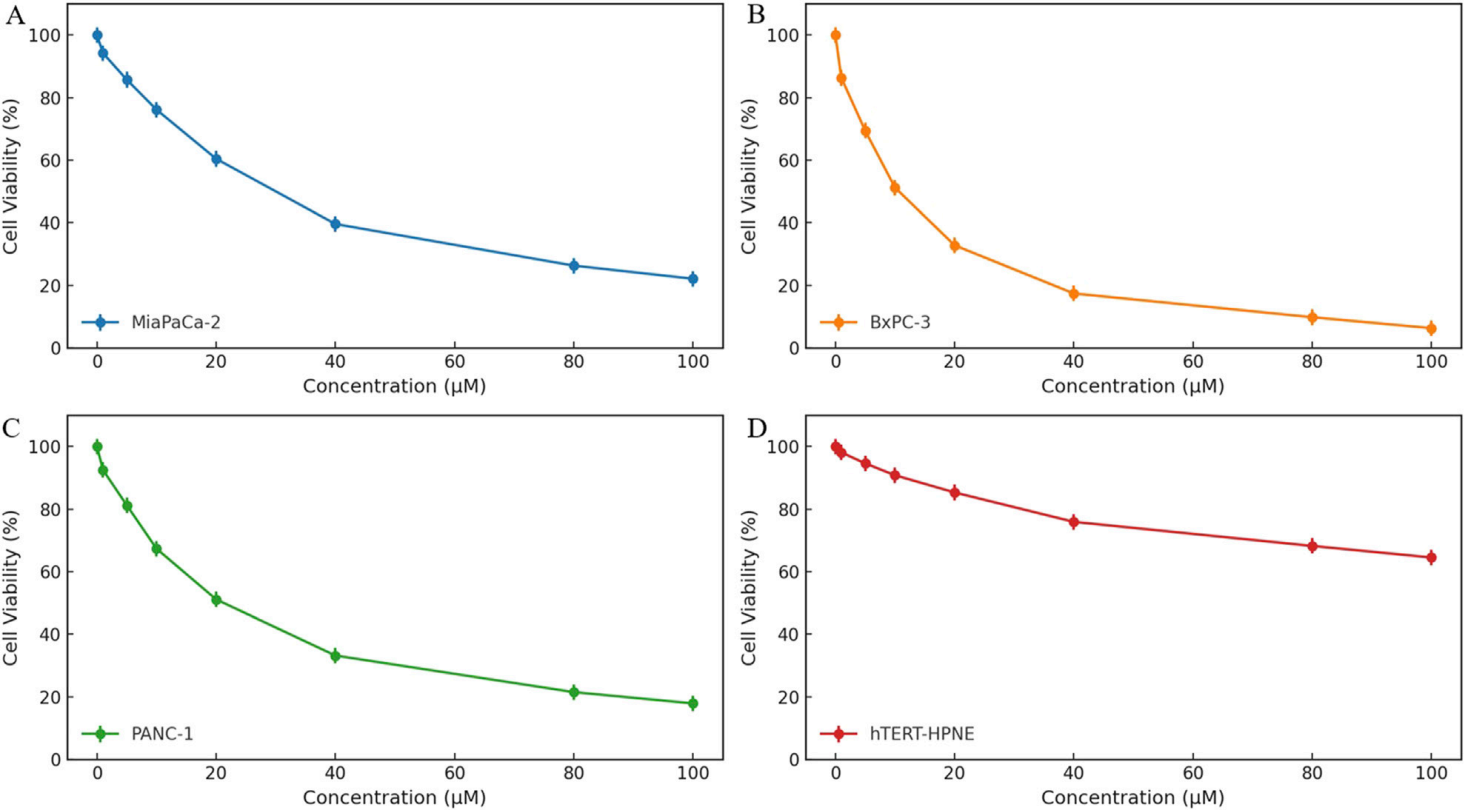
Cytotoxic effects of HIT101481851 on three pancreatic cancer cell lines (MiaPaCa-2, BxPC-3, and PANC-1) and the normal pancreatic epithelial cell line hTERT-HPNE. Cells were treated with HIT101481851 for 24 h and subjected to CCK-8 assay. The X-axis indicates compound concentration (0–100 μM), and the Y-axis represents relative cell viability (%). The 0 μM group was treated with 5% DMSO as vehicle control. Each point represents the mean ± SD (n = 3).

To investigate the antiproliferative potential of HIT101481851, a cell viability assay was conducted using the CCK-8 method across three human pancreatic cancer cell lines (MiaPaCa-2, BxPC-3, and PANC-1) and a non-tumorigenic human pancreatic ductal epithelial cell line (hTERT-HPNE), as shown in Figures 6A–D. Cells were exposed to a concentration gradient of HIT101481851 (0–100 μM) for 24 h. The 0 μM group was treated with 5% DMSO and served as the negative control. Optical density (OD) values were measured with a microplate reader and normalized to calculate relative cell viability.

The results revealed a clear dose-dependent cytotoxic effect of HIT101481851 in all three pancreatic cancer cell lines, with differential sensitivity. Among them, BxPC-3 exhibited the highest sensitivity, with cell viability reduced to 6.3% at 100 μM and an IC_50_ value of 27.3 μM. PANC-1 and MiaPaCa-2 showed reduced viability of 17.9% and 22.1%, with IC_50_ values of 33.8 and 39.5 μM, respectively, indicating comparatively lower sensitivity. These data suggest a moderate level of cytotoxic potency under short-term exposure.

In contrast, the non-cancerous hTERT-HPNE cells displayed substantially higher tolerance, retaining 64.5% survival at the highest tested concentration, indicating a degree of tumor-selective toxicity. The dose–response curves, along with standard deviation error bars, confirm the reproducibility and robustness of the experimental results.

## 4 Discussion

In this study, we employed a multi-step structure-based virtual screening and molecular dynamics refinement approach to identify HIT101481851 as a novel small-molecule inhibitor targeting PKMYT1, with potential therapeutic relevance in PDAC. Mechanistically, PKMYT1 functions as a dual-specificity kinase that inhibits CDK1 by phosphorylating THR-14 and TYR-15, thereby enforcing the G2/M checkpoint to prevent premature mitotic entry under DNA damage or replication stress (Asquith et al., 2020). Tumor cells with TP53 mutations or elevated replication stress—such as those harboring co-occurring KRAS and TP53 mutations, frequently seen in PDAC—become increasingly reliant on PKMYT1-mediated checkpoint control (Chen et al., 2022; Tornesello, 2025). In such genetic contexts, PKMYT1 inhibition can force mitotic entry with unresolved DNA damage, inducing mitotic catastrophe and tumor cell death (Xu et al., 2025). Our *in vitro* assays confirmed that HIT101481851 exhibits dose-dependent cytotoxicity in PDAC cell lines while sparing non-tumorigenic epithelial cells, supporting its selective antitumor potential through synthetic lethality.

To discover inhibitors with high conformational adaptability, we constructed pharmacophore models based on four distinct PKMYT1 crystal structures (PDB IDs: 8ZTX, 8ZU2, 8ZUD, 8ZUL). From a pool of 1.6 million molecules, 130 consensus hits were selected by pharmacophore matching and docking. HIT101481851 emerged as a top candidate due to favorable docking scores and consistent binding across three of the four conformations. Molecular dynamics simulations validated its stable interactions (Xu et al., 2024) with conserved ATP-binding residues including VAL-124, SER-193, PHE-240, and CYS-190, with instability observed only in 8ZU2, likely due to an unfavorable protein conformation (Tomović Pavlović et al., 2024). Interestingly, HIT101481851 showed a recurring binding pattern with PHE-252 and ASN-238, suggesting a potentially underexplored sub-pocket for future optimization.

Although HIT101481851 exhibits a novel chemical scaffold and favorable conformational adaptability across multiple PKMYT1 structures, its *in vitro* potency remains moderate, with IC_50_ values ranging from 27 to 40 μM in PDAC cells. While this is higher than the reported potency of certain co-crystallized ligands, such as compound 8ma or RP-6306, it is important to note that our screening strategy prioritized structural compatibility and dynamic binding stability across several PKMYT1 conformations rather than optimization for binding affinity alone. The crystal ligands found in available PKMYT1 structures were often obtained through medicinal chemistry refinement cycles, with optimized interactions targeting catalytically essential residues such as ASP-251 and TYR-121. In contrast, HIT101481851 was identified from a single-round virtual screening pipeline using semi-flexible docking and consensus pharmacophore filtering, without iterative structure–activity relationship (SAR) optimization (Ronco et al., 2017; Shaveta and Singh, 2014; Li et al., 2024). Therefore, its modest IC_50_ may reflect its early-stage status as an unrefined scaffold. Nonetheless, its unique binding orientation, particularly the consistent engagement with residues such as CYS-190 and PHE-252—regions not prominently exploited by the original co-crystallized inhibitors—suggests that HIT101481851 may occupy an alternative subpocket of the active site. This offers a new starting point for chemical elaboration aimed at accessing underutilized regions within the PKMYT1 catalytic domain, which could potentially improve selectivity and affinity in future analogs.

From a pharmacological perspective, HIT101481851 demonstrated favorable drug-likeness, including high predicted gastrointestinal absorption, non-P-glycoprotein substrate status (Ximenez et al., 2024), acceptable synthetic accessibility, and the absence of PAINS or Brenk structural alerts (Yadav et al., 2024). These characteristics suggest that the compound, in its current form, possesses core properties compatible with oral administration and drug development. However, it also exhibited clear pharmacokinetic liabilities. Most notably, its predicted aqueous solubility is poor (logS < −6.0), which may limit bioavailability or necessitate specialized formulation strategies. Moreover, HIT101481851 was predicted to inhibit multiple cytochrome P450 isoforms, including CYP1A2, 2C9, 2C19, 2D6, and 3A4 (Tamemoto et al., 2023). This broad-spectrum CYP inhibition raises concerns regarding drug–drug interactions, hepatic metabolism, and off-target toxicities—factors that are particularly relevant for PDAC patients undergoing multi-agent chemotherapy (Lin and Lu, 1998). Additionally, the compound is predicted to cross the blood–brain barrier, a feature that, while unnecessary for PDAC therapy, introduces potential safety concerns related to CNS exposure and off-tumor kinase inhibition.

These ADMET liabilities are characteristic of early-stage hits and provide valuable insights for future optimization (Ferreira and Andricopulo, 2019). The observed CYP450 inhibition may stem from structural features such as high aromaticity, planarity, or electron-rich motifs capable of π–π stacking or heme iron coordination—properties known to correlate with CYP promiscuity (Deodhar et al., 2020). Medicinal chemistry efforts should focus on reducing lipophilicity, disrupting planarity, and incorporating polar or solubilizing groups to minimize metabolic liabilities and enhance solubility. For instance, modifying the aromatic core to introduce hydrogen bond donors or adjusting the placement of heteroatoms could improve aqueous solubility without compromising target affinity (Walker, 2017). As for the compound’s CNS permeability, while it could be exploited in the context of CNS malignancies like glioblastoma where PKMYT1 is also overexpressed, this property necessitates neurotoxicity evaluation during preclinical development. Ultimately, addressing these ADMET challenges—through structure refinement, metabolic profiling, and early *in vivo* pharmacokinetic testing—will be essential to transform HIT101481851 from a structurally promising hit into a clinically viable lead compound (Cerny et al., 2020; Chhatrapati Bisen et al., 2023).

Clinically, the most advanced PKMYT1 inhibitor, RP-6306, is undergoing multiple phase I/II trials, often in combination with gemcitabine, carboplatin, or irinotecan-based chemotherapy (Szychowski et al., 2022). These studies have shown that PKMYT1 inhibition is particularly effective in tumors with CCNE1 amplification, FBXW7 loss, or PPP2R1A mutations—all genomic alterations that converge on heightened replication stress or G2 checkpoint dependence (Zhong and Virshup, 2024; Haesen et al., 2016). Although we did not stratify HIT101481851s efficacy by such molecular markers, its mechanism of action suggests it may be especially efficacious in KRAS/p53-mutant PDAC, which accounts for over 60% of pancreatic cancers. Thus, HIT101481851 offers a rational starting point for further optimization, both in potency and pharmacokinetic behavior, to enable its use in biomarker-driven precision oncology.

In conclusion, HIT101481851 represents a novel PKMYT1-targeting chemotype that demonstrates conformationally stable binding and tumor-selective cytotoxicity in PDAC models. Although its potency and drug-like properties require further improvement, the compound forms a robust scaffold for iterative medicinal chemistry efforts. Future directions include chemical modification to enhance aqueous solubility and reduce CYP inhibition, *in vitro* validation of CDK1 dephosphorylation as an on-target effect, and *in vivo* efficacy testing in genetically stratified models, particularly KRAS/p53 co-mutant PDAC. Given the growing evidence of PKMYT1 as a synthetic lethality node in replication-stressed cancers, HIT101481851 may contribute to the next-generation of precision therapeutics targeting cell cycle vulnerabilities.

## 5 Conclusion

PKMYT1 is a well-established therapeutic target in pancreatic cancer due to its critical role in regulating cell cycle progression and tumor proliferation. In this study, we employed a structure-based drug discovery strategy leveraging multiple co-crystal structures of PKMYT1 to identify potent inhibitors. Through pharmacophore modeling, molecular docking, virtual screening, and molecular dynamics simulations, HIT101481851 was identified as the most promising candidate. This compound exhibited robust binding stability across multiple PKMYT1 conformations, conserved interaction patterns with key residues such as CYS-190 and PHE-240, and favorable ADMET properties. Importantly, HIT101481851 displayed dose-dependent cytotoxicity in pancreatic cancer cell lines while sparing non-tumorigenic cells, suggesting selective antitumor activity. Collectively, these results support HIT101481851 as a potential lead compound for further development of PKMYT1-targeted therapies against pancreatic cancer.

## Data Availability

The original contributions presented in the study are included in the article/Supplementary Material, further inquiries can be directed to the corresponding author.
